# Point of care randomization (POCR) engine: demonstration of a transportable clinical informatics technology that improves health systems’ ability to conduct embedded clinical trials

**DOI:** 10.1093/jamiaopen/ooag209

**Published:** 2026-10-28

**Authors:** Catherine Sarkisian, Khalda Ibrahim, Sitaram Vangala, Chad Wes Villaflores, Eric M Cheng, William Turner, Richard K Leuchter, Amy Machado, Julia Tabar, Jade A Verdeflor, Jory Purvis, Alina Goncharova, Mark J Pletcher

**Affiliations:** Division of General Internal Medicine and Health Services Research, David Geffen School of Medicine at UCLA, Los Angeles, CA 90049, United States; Department of Pathology and Laboratory Medicine, University of California Los Angeles, Los Angeles, CA 90095, United States; Division of General Internal Medicine and Health Services Research, David Geffen School of Medicine at UCLA, Los Angeles, CA 90049, United States; University of California Los Angeles David Geffen School of Medicine Department of Medicine Statistics Core, Los Angeles, CA 90024, United States; Division of General Internal Medicine and Health Services Research, David Geffen School of Medicine at UCLA, Los Angeles, CA 90049, United States; Division of Medical Informatics, David Geffen School of Medicine at UCLA, Los Angeles, CA 90095, United States; Division of General Internal Medicine and Health Services Research, David Geffen School of Medicine at UCLA, Los Angeles, CA 90049, United States; Division of General Internal Medicine and Health Services Research, David Geffen School of Medicine at UCLA, Los Angeles, CA 90049, United States; Health Information Technology, Information Services & Solutions, Medical Informatics, University of California Los Angeles, Los Angeles, CA 90095, United States; Health Information Technology, Information Services & Solutions, Medical Informatics, University of California Los Angeles, Los Angeles, CA 90095, United States; Health Information Technology, Information Services & Solutions, Medical Informatics, University of California Los Angeles, Los Angeles, CA 90095, United States; School of Medicine Technology Services, University of California San Francisco, San Francisco, CA 94143, United States; Research Informatics, University of California San Francisco, San Francisco, CA 94143, United States; Department of Epidemiology and Biostatistics, University of California San Francisco, San Francisco, CA 94143, United States

**Keywords:** MeSH, electronic health records, randomized controlled trials as topic, clinical decision support systems, quality improvement, pragmatic clinical trials as topic, medical informatics, workflow

## Abstract

**Objective:**

We describe a new point of care randomization (POCR) engine developed at UCSF and implemented at UCLA that supports randomization of patients, encounters, clinics, or any other “object” in Epic, with programmable ratios, strata, and randomly varying block sizes.

**Materials and Methods:**

The feature, programmed in Caché, uses Epic rule infrastructure to implement inclusion criteria and generate and use blocks of randomization assignments. Configuration details, randomization assignments, and context information (including stratification variables) are stored in custom master files in Chronicles and exported to Clarity (Epic’s reporting database). As a demonstration use-case, we conducted a pragmatic RCT of a provider-facing alert for short-interval (30-day) repeat HbA1c orders at UCLA Health. At the instant providers initiated a repeat order, the POCR engine silently enrolled and randomized patients, stratified by age and previous HbA1c, with randomly varying block sizes.

**Results:**

During the 3-month study, 333 patients were enrolled. The total number of orders signed in the intervention arm (primary outcome) was 18/172 (10%) vs 104/161 (65%) in the control arm (Incidence Rate Ratio 0.16, 0.95 CI, 0.10-0.27).

**Discussion:**

We successfully transported and deployed a new technology with the capability to instantaneously perform automated stratified block randomization. By performing eligibility assessment, enrollment, and assignment using programmable ratios, strata, and randomly varying block sizes, the POCR engine preserves internal validity while minimizing disruption to care, representing a substantive advance in informatics infrastructure for embedded randomized trials.

**Conclusion:**

The POCR engine is a transportable clinical informatics technology that transforms health systems’ ability to conduct pragmatic clinical trials within the EHR.

## Introduction

The widespread adoption of electronic health records (EHRs) offers tremendous opportunity to improve healthcare delivery and patient outcomes. Accordingly, measuring the impact of EHR interventions is a timely and active area of translational informatics research. Typically, health systems and their clinical informatics and quality improvement teams implement EHR interventions that “make sense” on the grounds that the mechanisms by which they could improve outcomes are bioplausible. But these interventions are rarely tested in randomized controlled trials (RCTs) to prove that they are effective and do not cause unintended harms.^1^ Common examples include provider-facing point-of-care decision support alerts and patient-facing health maintenance reminders. While the biomedical literature is full of pre-post analyses of these types of EHR interventions, it is difficult to confirm whether these projects are truly successful or whether pre-post differences represent secular trends and/or regression to the mean.^2^

Seeking causal inference through randomization, some research teams utilize “pseudorandomization” with surrogate methods such as day of week or odd/even medical record number.^3^ Pseudorandomization does not allow for techniques—such as block or stratified randomization—that facilitate balanced assignment within treatment groups; it makes the randomization assignment for any given unit predictable and potentially unblinded; and it may build in structural group differences (eg, patients who present on Sunday may be different than patients presenting on Monday). True randomization with stratification and blocking currently requires cumbersome processes—for example, randomizing potentially eligible patients or providers outside the EHR with a random number generator and then assigning their study arm prior to an upcoming encounter in which the EHR intervention would be triggered. Workaround processes like these fix arm assignments at the beginning of the intervention (such as when an alert “goes live”), even though enrollment may unfold over months. Someone randomized *ex ante* may never actually trigger the action of interest (eg, by ordering a certain medication or lab test), and individuals who were not already randomized *ex ante* (eg, new hires or newly empaneled patients) are absolutely excluded from the study.

Over the past decade, substantial progress has been made toward facilitating real-time randomization within the EHR. In 2017, a team from Yale presented at Epic XGM a method for automatic randomization at the patient level within Epic using an internal random number rule with concealed allocation.^4^ This method has been adopted by several other institutions since, including NYU, which in 2021 published a tip sheet on how to use this method.^5^ Recently, Epic has created standard functionality to randomize by patient, user, and patient encounter without requiring custom code/additional licenses. Unfortunately, none of these existing options allow for stratified block randomization; stratified randomization is considered state of the art for randomized trials because it helps prevent chance imbalances in key prognostic variables that could otherwise reduce precision or threaten validity.^6^^,^^7^

The purpose of this article is to: (1) describe a new clinical informatics technology developed at UCSF—the point of care randomization (POCR) engine–that supports real-time randomization of patients, encounters, clinics, or any other “object” in Epic, with programmable ratios, strata, and randomly varying block sizes; (2) describe the application of the transportable POCR engine with a demonstration use-case of a pragmatic randomized trial of a provider-facing alert for short-interval HbA1c orders at UCLA Health; and (3) provide practical implementation details regarding the UCLA adoption experience. The randomized trial serves as a demonstration use-case to validate feasibility and impact, rather than as the primary scientific contribution of this article. This POCR engine could be disseminated to numerous health systems across the US using Epic systems with the capacity to integrate it; it also serves as an example to inspire other creative informaticist scientists to innovate and test customized solutions against the numerous challenges facing EHR research.

## Methods

In 2020, the Clinical and Translational Sciences Institute (CTSI) at the University of California, San Francisco (UCSF) developed a new clinical informatics technology (“feature”) to automate randomization within the EHR. The feature, programmed in Caché (Epic’s database engine layer that stores and serves live EHR data), uses Epic rule infrastructure to implement inclusion criteria, and generate and use blocks of randomization assignments. Configuration details, randomization assignments, and context information (including stratification variables) are stored in custom master files in Chronicles (Epic’s core operational database, where real-time clinical and administrative data is stored and retrieved) and exported to Clarity (Epic’s reporting database). The system supports randomization of patients, encounters, providers, clinical units, or any other type of “object” in Epic, with programmable randomization ratios, strata, and randomly varying block sizes to ensure balance and unpredictability. UCSF uses this Point-of-Care Randomization (POCR) engine for randomized trials of interventions in their EHR.^8–10^

University of California, Los Angeles (UCLA), though also part of the larger University of California, operates as an independent health system supported by its own instance of Epic that is separate from UCSF’s instance. UCLA Health has a long-standing commitment to being a learning health system.^11^ As part of this mission, the health system supports UCLA Healthcare Value Analytics and Solutions (UVAS)—a research team based in the School of Medicine that works closely with the Chief Medical Information Officer, Dr. Eric Cheng, and UCLA Informatics Systems and Solutions (ISS) to provide timely analyses to inform health system operations and improve healthcare delivery. In 2023, UCLA Healthcare Value Analytics and Solutions, UCLA Informatics Systems and Solutions, and the UCLA Office of Health Informatics and Analytics (OHIA) partnered with UCSF to deploy the POCR engine at UCLA Health.

**EHR software vendor** 

Both UCSF Health and UCLA Health independently manage their own instance of Epic for their main EHR platform. UCSF’s customized implementation of Epic is called APeX (Advancing Patient-Centered Excellence), and UCLA’s customized implementation is called CareConnect.

**Deployment of the POCR engine** 

The UCSF APeX-Enabled Research team provided demonstrations to the UCLA Healthcare Value Analytics and Solutions, the UCLA Informatics Systems and Solutions, and the Office of Health Informatics and Analytics teams, with step-by-step instructions on implementation of the randomization infrastructure within Epic. The UCSF team shared specific custom code and were available to answer questions as needed. The UCLA Health team deployed the UCSF POCR engine following these steps (#1 to 4 for installation and #5 to 9 for implementation). Pseudocode is provided in the Supplementary material. See Figure 1.

**Figure 1.**
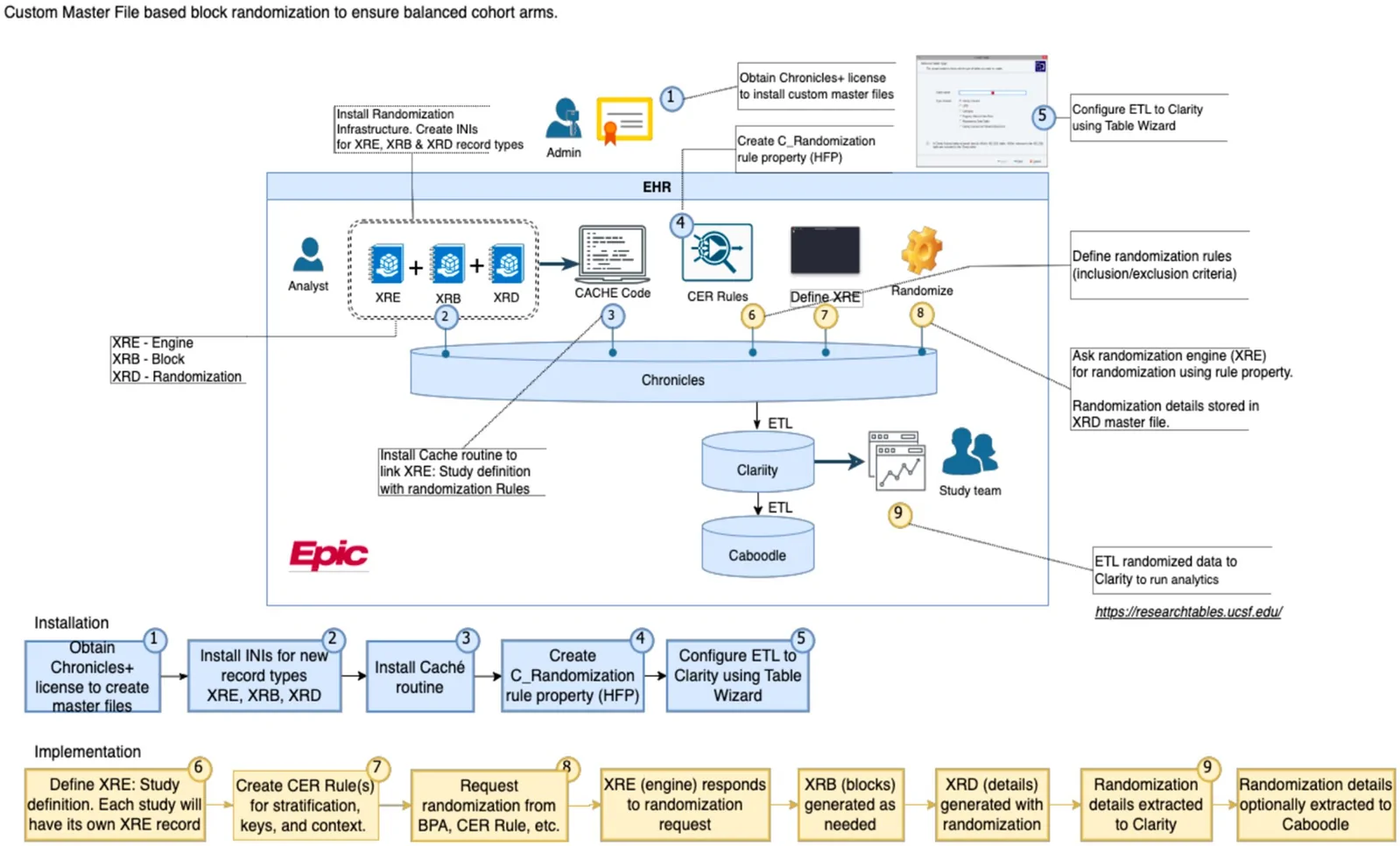
POCR engine. This figure shows the point-of-care randomization engine infrastructure. Installation steps (blue) are completed once per environment (eg, testing or production) and require a Chronicles + license, installation of Cache routines, and configuration of INIs (Master Files) for XRE (Engine Master File), XRB (Block Master File), and XRD (Randomization Master File). (XRE, XRB, and XRD are Epic technical object prefixes; XRE often refers to an Epic extract or export-related object, while XRB often refers to a build or binary-related object, and XRD often refers to a definition, descriptor, or data-related object.) Configuration of the ETL (extraction-transformation-load) processes allows the data to be loaded into the appropriate Clarity table. Implementation steps (yellow) are performed for each randomization project and use XRE, XRB, and XRD to define study-specific configurations. CER rule(s) are defined and implemented for each randomization project.

1. Purchase Chronicles + license to create master files in Chronicles. (Chronicles is Epic’s proprietary database management system designed specifically to process clinical, operational, and transactional data in real time for every patient encounter.)
2. Install randomization engine infrastructure, creating new Master Files for engine (XRE), block (XRB), and randomization (XRD) types. (XRE, XRB, and XRD are Epic technical object prefixes; XRE often refers to an Epic extract or export-related object, while XRB often refers to a build or binary-related object, and XRD often refers to a definition, descriptor, or data-related object.)
3. Install Caché code to utilize the new master files and generate/use randomizations.
4. Configure extraction-transformation-load processes to Clarity using Table Wizard.
5. Create randomization rule property for a particular RCT.
6. Define inclusion and exclusion randomization rules and other configuration specifics for a particular RCT.
7. Save configuration details for the RCT in XRE (randomization context, stratification, block size multiplier, etc).
8. Trigger randomization events using rule property; randomization assignments are made according to stratified blocked assignments stored in XRB (new blocks of assignments are generated as needed by the program); and then the randomization assignment and contextual information including strata variables are stored in the XRD master file.
9. The randomization assignment is then available for use (immediately and subsequently) as a component of a rule.
10. Data from XRE, XRB, and XRD are automatically extracted to Clarity (a separate relational data warehouse built by Epic for advanced reporting, analytics, and research) and can be optionally extracted to Caboodle (Epic’s enterprise data warehouse).

Completion of this system-level infrastructure, including implementing the first use-case randomized trial described below, took approximately 340 hours of UCLA staff time.

### Demonstration use-case at UCLA health: pragmatic RCT of provider-facing alert for low-value repeat HbA1c testing

#### Overview

As a demonstration use-case for the POCR engine at UCLA, UVAS collaborated with the Laboratory Stewardship Task Force on an initiative to reduce redundant testing that does not improve care. We conducted a pragmatic quality improvement RCT of provider-facing clinical decision support (CDS) designed to discourage inpatient orders for hemoglobin A1c (HbA1c) within 30 days of prior HbA1c testing (which we term “short-interval HbA1c orders”); repeating HbA1c testing within 30 days is generally not indicated.^12^ Whenever providers initiated an order for repeat HbA1c testing, eligible patients were silently enrolled and randomized within the EHR using the POCR Engine. For a random half of patients, their providers were immediately shown the interruptive alert (intervention arm); the other half of patients were silently enrolled with no alert fired (control arm—care as usual).

#### Study design and setting

This was a pragmatic, parallel-arm RCT at a large urban academic health system of three non-psychiatric adult hospitals with a combined bed count of approximately 1,000. The unit of randomization was the patient (see below). We carefully considered various study designs, including randomization at the level of the provider, especially as randomizing patients could introduce a risk of contamination across study arms when providers exposed to the intervention early in the study could subsequently see a patient in the control arm and might change their ordering behavior based on their previous exposure to the intervention. We decided that because this risk was small, would occur rarely, and would bias the study toward the null hypothesis, it was outweighed by the benefit of increased power and associated the short study time-period with patient-level randomization.

#### Inclusion criteria

All inpatients were eligible and silently enrolled if they were age 18 years or older and their provider was initiating an order for HbA1c testing within 30 days of a previous HbA1c test result or while results of a recent HbA1c order were still pending.

#### Intervention and intervention implementation

The intervention consisted of a provider-facing CDS designed to discourage short-interval HbA1c orders. The CDS is shown in Figure 2. Providers had the option either to override the alert and sign their initiated HbA1c order or to acknowledge the alert and not sign the order.

**Figure 2.**
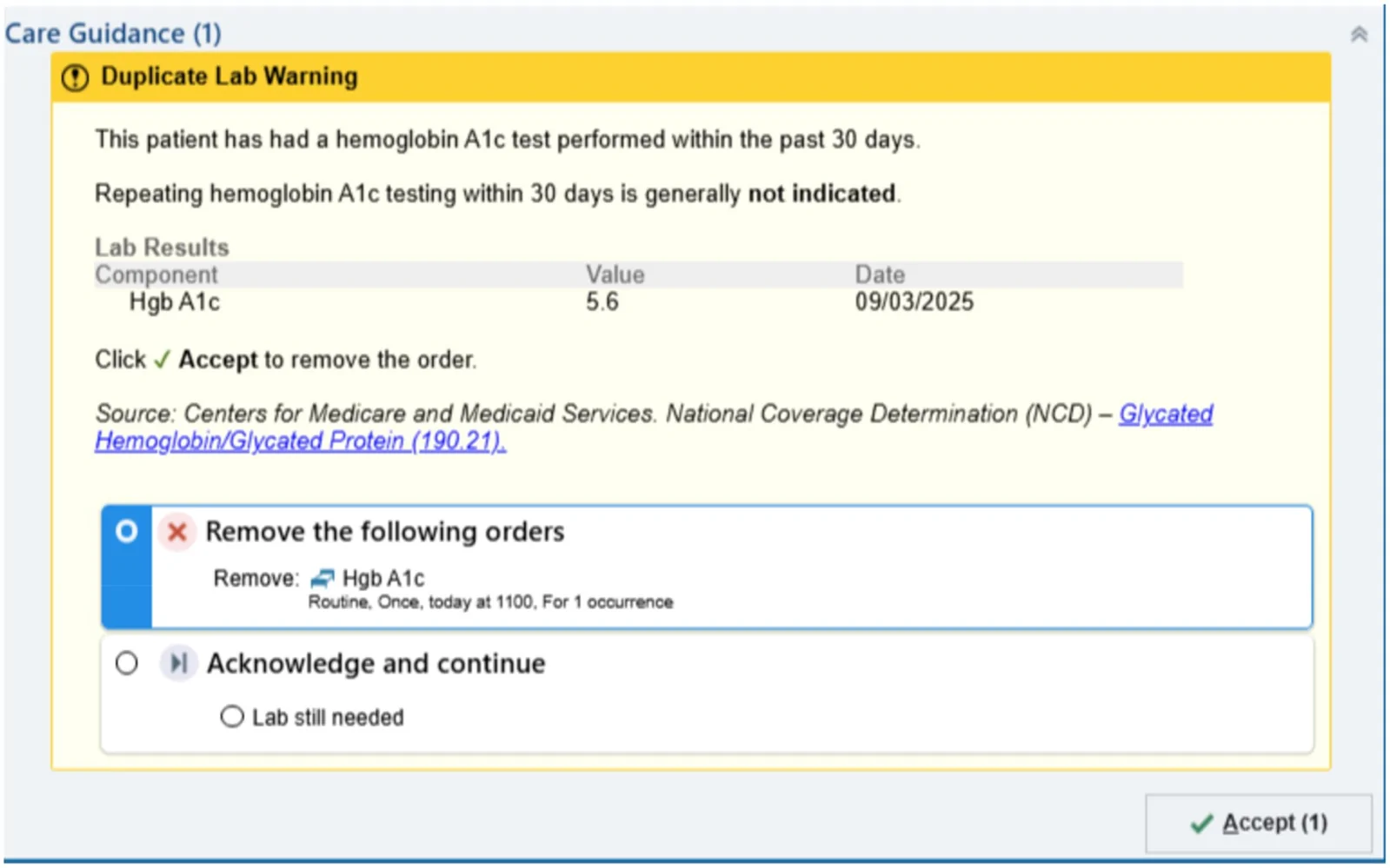
Provider-facing interruptive alert. This figure shows the provider-facing interruptive CDS alert displaying the patient’s most recent HbA1c value and test date. By default, the pending HbA1c order is removed, with the option for the provider to override the alert and proceed with testing. © 2026 Epic Systems Corporation.

Through an interdisciplinary iterative design process, we developed the alert to display the date and results of HbA1c testing performed in the previous 30 days (or in the case of pending results, to report the time the test was ordered). We hypothesized that if busy providers taking care of adult inpatients: (1) were notified in real time that their patient had had HbA1c testing performed recently; (2) were automatically provided with the date and results of that testing (or told that the result was pending); and (3) were advised that repeat testing within 30 days is “generally not recommended,” they would not sign and finalize their initiated HbA1c orders.

Unlike UCLA Health’s usual practice when launching a new informatics alert, no system-wide electronic mail education to all providers was delivered.

#### Randomization and blinding

The randomization of patients within each arm was stratified based on patient age (18 to < (up to but not including) 55 years, 55+) and most recent HbA1c values (<6.5%, 6.5%-8%, >8%). We stratified randomization to ensure balance between study arms on age and HbA1c level because these variables could impact the likelihood of a provider responding to the intervention. As noted in the Introduction, stratified randomization is considered state of the art for randomization because it helps prevent chance imbalances in key prognostic variables that could otherwise reduce precision or threaten validity.^6,7^ We randomly generated blocks of 4, 6, or 8 patients within each strata. At the instant when a provider initiated an order for a HbA1c test for a study-eligible patient, the POCR engine enrolled the patient and assigned the patient to either the intervention arm or the control arm. Once a patient was randomized, they stayed in that same study arm for the duration of the study, even if they had a subsequent eligible encounter. Providers and patients were not notified about the study. Providers in the intervention arm were shown the alert but were not aware that it was part of a study or that they had been randomly assigned to an intervention arm.

#### Study outcomes

The primary outcome was the number of signed HbA1c orders within 30 days of a prior HbA1c result or a pending HbA1c order during the observed encounters. Secondary outcomes included frequency of order cancelation and alert overrides in the intervention arm.

#### Data collection methods

Data was extracted from UCLA’s Clarity database.

#### Statistical analyses

The project analyst compared the rate (orders per randomized patient) between study arms of the primary outcome—the number of signed HbA1c orders—using Poisson regression. We calculated the incidence rate ratio (IRR), the difference in rates (number of signed HbA1c orders per randomized patient) between the intervention and the control arm. Analyses were performed using Python.

#### Sample size analyses

Based on previous ordering patterns, we estimated that the randomization would trigger 32 times per week, with 416 patients enrolled over 3 months and 208 in each study arm, providing 80% power to detect a 25-percentage point difference in the number of short-interval HbA1c orders with an alpha of 0.05.

#### Ethical issues

Recognizing that randomization in itself does not deem a project to be research involving human subjects as defined by DHHS and FDA regulations,^13^ the Institutional Review Board (IRB) of the UCLA determined that “the proposed activity is not research involving human subjects as defined by DHHS and FDA regulations” and that “IRB review and approval by this organization is not required.” With a goal of transparency and advancing shared learnings across institutions, this study was publicly registered with ClinicalTrials.gov (NCT06993805) on April 30, 2025.

## Results

The study took place over the pre-planned timeframe of 3 months (May 12, 2025-August 12, 2025). During the study period, 333 patients were enrolled and randomized, with 172 in the intervention arm and 161 in the control arm (see Figure 3). None of the patients had more than one eligible encounter during the study period. Patient characteristics are shown in Table 1.

**Figure 3:**
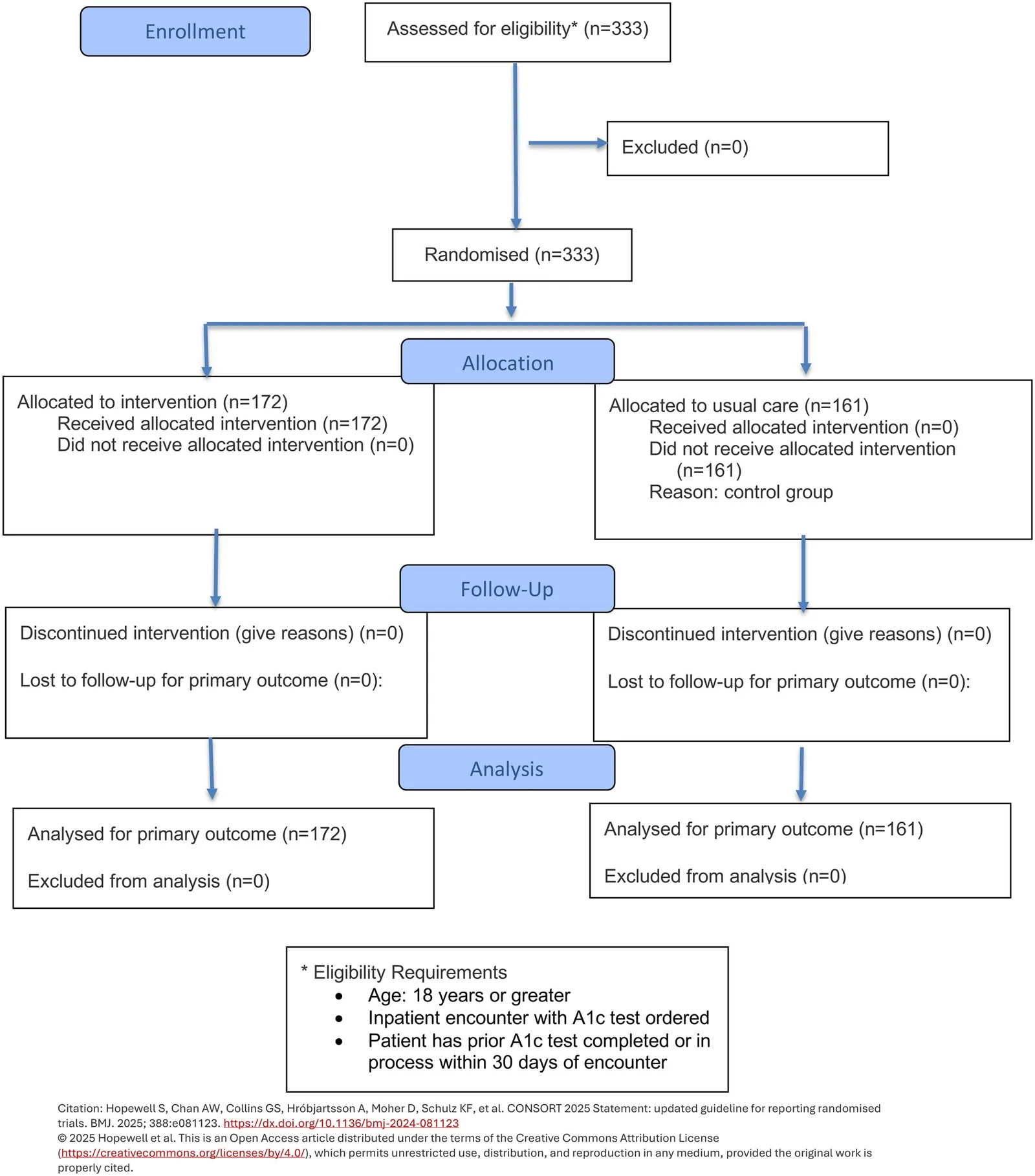
CONSORT 2025 flow diagram. CONSORT diagram showing the flow of eligible inpatient encounters through enrollment, randomization, allocation, follow-up, and analysis. Flow diagram of the progress through the phases of a randomized trial of two groups (ie, enrollment, intervention allocation, follow-up, and data analysis).

**Table 1.** Patient characteristics.

	Total *N* = 333	Intervention arm *N* = 172	Control arm *N* = 161
**Age, mean years (SD)**	62 (16)	62 (15)	62 (17)
**Male, *n* (%)**	213 (64)	112 (65)	101 (63)
**Self-reported race/ethnicity, *n* (%):**			
**Asian**	36 (11)	13 (8)	23 (14)
**Black or African American**	44 (13)	21 (12)	23 (14)
**Latino/Hispanic**	90 (27)	53 (31)	37 (23)
**White**	119 (36)	62 (36)	57 (35)
**Other**	24 (7)	10 (6)	14 (9)
**Unknown or not reported**	20 (6)	13 (8)	7 (4)
**Last HbA1c, mean % (SD)**	6.5 (1.7)	6.6 (1.8)	6.5 (1.6)

Mean age was 62.2 years, with 36.0% female participants. Mean age and last HbA1c results were nearly identical between study arms, indicating that the stratified randomization achieved good balance.

There were 249 unique providers who had an encounter with an enrolled patient during the study period; 201 (81%) of these had only a single encounter. Among the 48 providers who had more than 1 encounter, 25 of these had at least 1 encounter with a patient in both arms.

The total number of signed orders in the intervention arm (primary outcome measure) was 18/172 (10%) vs 104/161 (65%) in the control arm (IRR 0.16, 0.95 CI, 0.10–0.27). The alert was initially dismissed 34 times, with providers selecting the button “lab still needed,” but in 16 of these 34 encounters, the order was never signed, resulting in the total of 18 orders in the intervention arm as reported above. In one case, the provider wrote a free text comment of “s/p pancreas transplant” as the reason the lab was still needed. Zero orders were canceled in the control arm.

## Discussion

We successfully deployed an innovative point-of-care randomization (POCR) engine—originally developed at UCSF—into UCLA Health’s EHR, to our knowledge making UCSF the first, and UCLA Health, the second health system in the nation with the capability to instantaneously perform automated stratified block randomization of patients, encounters, providers, clinical units, or any other type of “object” at the point-of-care or point-of-order. With the new ability to randomize with programmable ratios, strata, and randomly varying block sizes “on the fly” in real time within the EHR, our use-case RCT clearly and efficiently demonstrated that this CDS dramatically reduced redundant HbA1c orders. Because we were able to learn that the alert was so effective, when the pre-planned 3-month study period ended, the POCR engine was turned off; 100% of short interval repeat HbA1c tests on adult inpatients now trigger the alert, as well as short interval lipid panels and TSH orders. This is protecting our patients from unnecessary testing while saving our health system thousands of dollars every month including saving provider time spent reviewing results from unnecessary tests. Due to high demand for the POCR randomization engine by providers and researchers at UCLA Health, a new governance council has been established to prioritize resource allocation of this valuable tool, and to assist research teams with study design issues such as inclusion criteria, selection of randomization strategies, and key performance indicators.

Consistent with ethical frameworks for learning health systems,^14^ this study employed silent enrollment for a minimal-risk intervention integrated into routine clinical care. The POCR engine supports this approach by enabling stratified block randomization only at the moment of a clinically relevant action, without altering standard workflows or withholding care. By facilitating rigorous evaluation of EHR interventions while preserving clinical autonomy and operational feasibility, POCR aligns with widely endorsed principles that emphasize continuous learning, proportional oversight, and the ethical obligation to generate evidence to improve care.^15^

To sum up, the POCR engine has transformed our ability to conduct pragmatic RCTs with programmable ratios, strata, and randomly varying block sizes within the EHR, dramatically elevating the impact of our quality improvement initiatives by facilitating our ability to quickly and efficiently learn whether our interventions improve care and outcomes. This represents an important advance from prior approaches to randomization of EHR-based interventions which have generally relied on methods that compromise internal validity or workflow integrity, and none of which allow stratified block randomization. By performing eligibility assessment, enrollment, and assignment using state of the art randomization methods in real time and entirely within routine clinical workflows, the POCR engine preserves internal validity while minimizing disruption to care, representing a substantive advance in informatics infrastructure for embedded randomized trials.

### Limitations to the randomized trial

Because the unit of randomization was the patient, we cannot rule out contamination across study arms, and 25 of the 249 unique providers had an encounter with a patient in both arms during the study; this would bias the study to the null, however, making the large observed effect size even more notable. It is also important to acknowledge that external validity was limited; this CDS alert should be tested in other health systems with different cultures and patient and provider-mix. As noted in the Introduction, the randomized trial serves as a demonstration use-case to validate the feasibility and impact of this new technology rather than as the primary scientific contribution of this article.

### Challenges and considerations

Health system research teams considering using the POCR engine should consider several challenges and implications associated with implementation. As noted above, this requires purchasing the Chronicles + license and the staff time commitment was substantial. Because of the staff time requirement, one challenge the UCLA team faced was that implementation was initially delayed when the team was working on the annual Epic upgrade (with a hard deadline). As with all custom codes that are not part of Epic’s native software, Epic does not take into account potential impact that changes in their software may have on the code, so it must be manually tested locally before each upgrade, independently by someone other than the person making the code changes. Because the code relies minimally on Epic’s native code, however, this risk is small, and is mitigated with regression testing of the code base 2–3 months prior to each scheduled upgrade. It is also important to note that to date, application of the POCR engine has been limited to academic health systems using Epic as their EHR vendor. Plans to deploy the POCR engine at other health systems outside California are underway; investigators seeking to use the POCR engine should reach out to the UCSF team at ResearchSolutions@ucsf.edu.

## Conclusion

The POCR engine is transforming health systems’ ability to conduct rigorous pragmatic clinical trials and deliver clinical care within the EHR, marking an important advancement in the ability to generate high-quality, causal evidence within healthcare systems. This POCR engine could be disseminated to numerous health systems across the U.S. using Epic systems with the capacity to integrate it; it also serves as an example to inspire other creative informaticist scientists to innovate and test customized solutions to the numerous challenges facing EHR research.

## Supplementary Material

ooag209_Supplementary_Data

## Data Availability

The data will be made available from the corresponding author upon reasonable request and with appropriate institutional approvals. Requests for access will be reviewed to ensure compliance with institutional review board (IRB) requirements and data use agreements. Investigators seeking to use the POCR engine should reach out to the UCSF team at ResearchSolutions@ucsf.edu.
