# Supplementary material for: Point of care randomization (POCR) engine: demonstration of a transportable clinical informatics technology that improves health systems’ ability to conduct embedded clinical trials
Source: JAMIA Open. 2026 Oct 28;9(5):ooag209. doi: 10.1093/jamiaopen/ooag209 (PMC13716527; doi:10.1093/jamiaopen/ooag209)
Supplement: Supplementary file 1 [file ooag209_supplementary_data.docx]

Embedded Point-of-Care Randomization Engine

# 1. Overall Randomization Algorithm

```
Algorithm: Configurable Research Randomization Engine

INPUT:
    record_id
    record_data
    randomization_engine
    allow_new_randomization

    randomization_engine contains:
        randomization_type              // block or fixed
        randomization_keys              // variables identifying the unit
                                         // of randomization
        stratification_rule             // optional; determines stratum
        context_variables               // variables recorded as context
        number_of_arms
        block_size_multiplier
        allocation_ratio
        fixed_assignment_rule           // used for fixed assignment
        context_recording_frequency
        maximum_total_assignments       // optional
        maximum_assignments_per_stratum // optional

PROCEDURE Randomize(record_id, record_data, engine, allow_new_randomization):

    1. Evaluate the configured randomization keys using the current record data.

       IF any required randomization key cannot be evaluated:
           RETURN no assignment

    2. Combine the evaluated key values into a randomization-unit identifier.

       // The randomization keys identify the unit of randomization.
       // They do NOT determine stratum.

    3. Search for an existing randomization associated with this
       randomization-unit identifier.

       IF an existing randomization is found:
           IF context updates are enabled:
               update stored context according to the configured frequency
           RETURN the previously assigned treatment arm

    4. IF creation of a new randomization is not authorized:
           RETURN no assignment

    5. Generate a new assignment according to the configured method.

       IF randomization_type = BLOCK:
           RETURN CreateBlockRandomization(...)

       IF randomization_type = FIXED:
           RETURN CreateFixedRandomization(...)

       OTHERWISE:
           RETURN no assignment
```

An assignment is reused when the same unit of randomization is encountered again, rather than generating another assignment for that unit.

# 2. Block Randomization

```
PROCEDURE CreateBlockRandomization(record_id, record_data, engine,
                                   randomization_keys,
                                   randomization_key_values,
                                   randomization_unit_identifier):

    1. Determine stratum.

       IF a stratification rule is configured:
           evaluate the stratification rule using the current record data
           IF the stratum cannot be determined:
               RETURN no assignment
       OTHERWISE:
           use a common/default stratum

    2. Check whether another randomization may be issued for this engine
       and stratum.

       IF the overall or stratum-specific allocation limit has been reached:
           RETURN no assignment

    3. Retrieve the active randomization block for this engine and stratum.

       IF no active block exists:
           create a new randomized block
       ELSE IF the active block is exhausted:
           create a new randomized block

    4. Automatically advance the position within the active block.
       IF the block cannot be advanced:
           RETURN no assignment

    5. Select the treatment arm corresponding to the current block position.

    6. Evaluate the configured context variables.

    7. Create and persist a randomization record containing:
           - randomization engine
           - block identifier
           - stratum
           - assigned treatment arm
           - randomization-unit identifier
           - randomization keys and evaluated values
           - evaluated context variables

    8. Update assignment counts for the engine overall and current stratum.

    9. RETURN the assigned treatment arm
```

# 3. Generation of Randomized Blocks

```
PROCEDURE GenerateBlock(number_of_arms, block_size_multiplier,
                        allocation_ratio):

    1. IF block_size_multiplier is unspecified or zero:
           randomly select a multiplier from {2, 3, 4}

    2. Initialize an empty block.

    3. FOR each treatment arm:
           determine the arm's allocation ratio
           IF no ratio is specified for the arm:
               use a ratio of 1

           number_of_assignments = allocation_ratio_for_arm
                                   x block_size_multiplier

           add that number of assignments for the treatment arm to the block

    4. Randomly shuffle the assignments within the block.

    5. RETURN the randomized block
```

Example: with two treatment arms, a multiplier of 2, and a 1:1 allocation ratio, the block contains two assignments to each arm before shuffling. With a 2:1 ratio and multiplier of 2, it contains four assignments to the first arm and two to the second before shuffling.

# 4. Block Selection and Replacement

```
PROCEDURE GetActiveBlock(engine, stratum):

    1. Search for the active block associated with this engine and stratum.

    2. IF no active block exists:
           RETURN CreateNewBlock(engine, stratum)

    3. Load the current block.

    4. IF current_position < block_size:
           RETURN current block

    5. OTHERWISE:
           generate and activate a new randomized block for this engine
           and stratum
           RETURN new block
```

# 5. Fixed Assignment

```
PROCEDURE CreateFixedRandomization(record_id, record_data, engine,
                                   randomization_keys,
                                   randomization_key_values,
                                   randomization_unit_identifier):

    1. Retrieve the configured fixed-assignment rule.
       IF no fixed-assignment rule exists:
           RETURN no assignment

    2. Evaluate the fixed-assignment rule using the current record data.
       IF the resulting assignment is invalid:
           RETURN no assignment

    3. Check whether another assignment may be issued for this engine.
       IF the configured allocation limit has been reached:
           RETURN no assignment

    4. Evaluate the configured context variables.

    5. Create and persist a randomization record containing:
           - randomization engine
           - fixed treatment assignment
           - randomization-unit identifier
           - randomization keys and evaluated values
           - evaluated context variables

    6. Update assignment counts.

    7. RETURN the fixed treatment assignment
```

# 6. Context Capture and Repeated Encounters

Configured context variables are evaluated and stored when a new randomization is created. When the engine subsequently encounters a unit that has already been randomized, it returns the existing assignment rather than creating another one. Depending on configuration, it can also record updated context associated with that encounter.

```
PROCEDURE UpdateContextForExistingRandomization(record_id, record_data,
                                                existing_randomization,
                                                engine):

    1. IF context-recording frequency = INITIAL ONLY:
           RETURN without modification

    2. Evaluate the configured context variables using current record data.

    3. IF context-recording frequency = ON CONTEXT CHANGE:
           retrieve the most recently stored context
           compare the new context with the previously stored context
           IF context has not changed:
               RETURN without modification

    4. Create a new contextual record associated with the existing
       randomization.

    5. Store the newly evaluated context.
```

# 7. Allocation Limits

```
PROCEDURE IsNewRandomizationAllowed(engine, stratum):

    1. Retrieve maximum_total_assignments and
       maximum_assignments_per_stratum.

    2. IF neither limit is configured:
           RETURN TRUE

    3. IF either applicable limit explicitly permits zero assignments:
           RETURN FALSE

    4. Retrieve the number of assignments already issued by this engine.
       IF no assignments have previously been issued:
           RETURN TRUE

    5. IF a total limit is configured AND
          total_assignments >= maximum_total_assignments:
           RETURN FALSE

    6. IF a stratum-specific limit is configured:
           retrieve assignments previously issued within this stratum
           IF stratum_assignments >= maximum_assignments_per_stratum:
               RETURN FALSE

    7. RETURN TRUE
```

# 8. Conceptual End-to-End Flow

```
RANDOMIZATION REQUEST
        |
        v
Evaluate randomization keys
        |
        v
Identify unit of randomization
        |
        v
Has this unit already been randomized?
        | YES                         | NO
        v                             v
Retrieve existing assignment     Is new assignment authorized?
        |                             | NO -> return no assignment
Update context as configured          | YES
        |                             v
        |                    Determine randomization method
        |                         /             \
        |                      BLOCK           FIXED
        |                        |               |
        |                 Determine stratum   Evaluate fixed rule
        |                        |
        |                 Check allocation limits
        |                        |
        |                 Retrieve/create block
        |                        |
        |                 Select next assignment
        |                        |               |
        |                        +-------+-------+
        |                                v
        |                         Capture context
        |                                |
        |                         Persist assignment
        |                                |
        |                         Update metrics
        +----------------+---------------+
                         v
                RETURN TREATMENT ARM
```

# 9. Key Conceptual Distinctions

- **Randomization key:** Identifies the unit of randomization. The configured key or combination of keys determines whether the engine is encountering a unit that has already received an assignment. An existing unit receives its previously generated assignment rather than being randomized again.
- **Stratification rule:** Determines the stratum within which block randomization occurs. It is evaluated independently of the randomization key. Each stratum maintains its own sequence of randomized blocks.
- **Context variables:** Capture additional information associated with the randomization and subsequent encounters. They do not determine the identity of the randomization unit or, by themselves, its stratum.

These components can be configured independently, allowing the unit of randomization, stratification variables, and contextual variables to serve distinct methodological roles.
